# Pilot Study on the Effects of the Teaching Interpersonal Skills Program for Teens Program (PEHIA)

**DOI:** 10.3389/fpsyg.2022.764926

**Published:** 2022-02-11

**Authors:** Isabel Serrano-Pintado, María-Camino Escolar-Llamazares, Juan Delgado-Sánchez-Mateos

**Affiliations:** ^1^Departamento de Personalidad, Evaluación y Tratamiento Psicológicos, Universidad de Salamanca, Salamanca, Spain; ^2^Departamento de Ciencias de la Salud, Universidad de Burgos, Burgos, Spain; ^3^Departamento de Psicología Básica, Psicobiología y Metodología de las Ciencias del Comportamiento, Universidad de Salamanca, Salamanca, Spain

**Keywords:** PEHIA, interpersonal skills, adolescents, quasi-experiment, training program

## Abstract

**Background/Objective:**

Social skills are essential in adolescence, both for their relational dimension and for their influence on other areas of adolescent life, so it is essential to include Social skills in the formal education of students.

**Method:**

This paper presents the results of an experimental mixed factorial design pilot study in which an Interpersonal Skills Training Program for Adolescents (PEHIA^1^) was applied. The convenience sample consisted of 51 adolescents. An evaluation was carried out before and after the intervention, using the CEDIA (Adolescent Interpersonal Difficulties Assessment Questionnaire) and SAS-A (Social Anxiety Scale for Adolescents) questionnaires.

**Results:**

The mixed factorial ANOVA show significant differences in the overall measures and in most of the subscales of both questionnaires, indicating that PEHIA is effectiveness, at least in the short term.

**Conclusions:**

The results obtained in assertiveness, interpersonal relationships and public speaking suggest that the program is feasible and shows promising results in reducing anxiety. However, a larger scale study should be conducted.

## Introduction

Adolescence is a critical period characterized by the development of the cognitive competencies and skills necessary for independent functioning during adulthood (DeRosier and Thomas, 2019; Sánchez-Sansegundo et al., 2020). In this sense, social skills are essential not only for their relational dimension, but also for their influence on other areas of adolescent life, as they operate as a protective factor and constitute a protective health resource in early adolescence (Carmona and López, 2015; Olivares-Olivares et al., 2019).

Social skills are a set of interpersonal behaviors necessary to interact and relate effectively with others. They are predominantly behavioral skills that: (1) allow the adequate expression of feelings, wishes and opinions; (2) allow criticism to be handled adequately; (3) minimize interpersonal conflicts; and (4) enable relationships with others effectively and to mutual satisfaction. Social skills enable adequate adaptive social behavior and they are related to the academic, emotional and social well-being of children (Monjas and González, 2000; Caballo, 2005; Carmona and López, 2015; Peñalva-Vélez et al., 2020). Early ages are critical in the development of social skills, as they are learned through interaction with others. Therefore, it is essential to incorporate them into the school curriculum (Monjas et al., 2009; Oros and Fontana Nalesso, 2015; Núñez Hernández et al., 2018; Peñalva-Vélez et al., 2020).

Numerous research studies have shown the strong relationship between adequate social functioning and social, academic, and psychological adjustment in childhood, adolescence, and adulthood (Rosa et al., 2002), without which there is an increased risk of behavioral problems in interpersonal relationships, difficulties in psychological well-being, academic performance and a greater likelihood of disruptive behaviors (Carmona and López, 2015; Armada Crespo et al., 2020; Shinde et al., 2020). Learning these skills depends primarily on practice, training and refinement, and not so much on verbal instruction. It is essential to exercise and practice social-emotional skills and turn them into more adaptive response of the person's natural repertoire. At a theoretical level, the need to include them in education is justified, but in practice there is still a long way to go (Carmona and López, 2015; Salwa et al., 2019; Meyer et al., 2020).

Accordingly, in recent years there has been a significant increase in the number of interventions designed to promote adolescent social competence in educational contexts as part of their comprehensive education. Research shows that social skills training is effective in teaching socially adaptive behaviors (Rosa et al., 2002; Llinares et al., 2015; Cervera Rojas et al., 2019; van Loon et al., 2019).

It should be noted that during early adolescence, a significant transition takes place in relation to the educational context. It usually happens that, either at the beginning of secondary education (12–13 years), or when the second cycle of this section begins (15–16 years), there is a change from school to high school. This change can lead to a breakdown of the group of friends formed around class or extracurricular activities. This destructuring can have a negative impact on the adaptation to the new school situation. In addition, upon arrival at the high school, students tend to have a more active and participatory role, which means that they must make oral presentations in public. For example, they must present their point of view or opinion in front of the class or in a student assembly (Inglés Saura, 2017; Salwa et al., 2019; van Loon et al., 2019).

In Spain, Inglés Saura (2017) points out the scarce efforts made to improve the interpersonal skills of adolescents in the educational field. In order to respond to this need, Inglés Saura presents the Interpersonal Skills Training Program for Adolescents^2^ (PEHIA) (Inglés, 2003). PEHIA is a direct and systematic teaching program of interpersonal skills whose general objective is to promote adequate interpersonal relationships, prevent maladaptive problems and eliminate and/or reduce possible interpersonal difficulties during adolescence. The PEHIA comprises seven interpersonal skills, divided into seven sessions, plus an introductory session. These skills have been empirically selected, that is, the adolescents determined which skills, which stimulus-persons and which areas or social contexts presented the greatest difficulty for them (Méndez et al., 2002). In this way, the opinion of the adolescents was taken into account, a fact that significantly improves the social validity of the intervention (Hansen et al., 1998).

The PEHIA is designed to be applied to adolescents, 12–18 years of age, that have: no apparent problems; with internalizing problems (social isolation, social withdrawal, shyness, low self-esteem); externalizing problems (aggressiveness, hostility, etc); and in situations of social risk (internal students in juvenile centers, etc.). The program is applied in ordinary school classrooms. The duration of the sessions is approximately 1 h. It can be applied by a wide range of professionals (teachers, psychologists, pedagogues, psychopedagogues, social workers, educators, etc.) (Inglés Saura, 2017).

One of the most important characteristics of this program is its flexibility in terms of the material to be used, the activities to be carried out, the size of the group, the number of monitors, as well as the frequency, duration, schedule and framework of the sessions. However, the use of all the materials and activities of the program is recommended in order to achieve the objectives set (Inglés Saura, 2017).

Likewise, it is convenient to carry out at least one session per week. If possible, the training can take place twice a week, as long as the space between the two sessions is 2 or 3 three days, so that participants have enough time to do the homework that is included in each session. Also, it is advised that each of the sessions be dedicated to the teaching of a single skill (Inglés Saura, 2017).

The PEHIA focuses on teaching specific social behaviors (e.g., saying no or refusing, giving and accepting compliments) through the application of behavioral techniques (instructions, modeling, behavior rehearsal or role-playing, positive reinforcement, feedback or feedback and homework assignments) that the monitors can easily adapt to the particular dynamics of each group (Inglés Saura, 2017).

All sessions include a script for the monitor, where all the activities to be carried out are specified and detailed.

Most of the sessions, except the first, follow the following sequence (Inglés Saura, 2017):

1. Review of homework from the previous session.
2. Verbal instruction of the skill to be trained: 2.1. Skill definition; 2.2. Exhibition and dialogue; 2.3. Importance of skill for participants: advantages and disadvantages; 2.4. Behavioral Skill Steps.
3. Identification of interpersonal response styles.
4. Modeling, role-playing, positive reinforcement, and feedback.
5. Review of the session.
6. Homework.

Although this program is known and has been included in several reviews made on the different social skills training programs (Cavada Bedia and Serrano Pintado, 2015; Llinares et al., 2015; Rubiales et al., 2018), we have not found studies that analyze its effectiveness. Thus, we propose to resolve this situation with the present research, as has been done with similar programs.

For instance, Sánchez-Sansegundo et al. (2020) consider that, to date, one of the most widely implemented cognitive-behavioral programs is Reasoning and Rehabilitation (“Reasoning and Rehabilitation”—R&R), a cognitive skills program that aims to address cognitive deficits and improve social and emotional skills in the youth and adult population. The program includes a wide range of strategies to improve problem solving, social perspective taking, critical reasoning, empathy, and negotiation skills through the use of games, and practical skills and debates. The R&R program has a reduced version, R&R2 (Sánchez-Sansegundo et al., 2020). These authors evaluated the effectiveness of both programs and reported an improvement in self-esteem, social skills, empathy and problem solving. They also obtained evidence of their effects in the implementation of these programs in school settings in Spain, North America and Canada (Sánchez-Sansegundo et al., 2020).

Hence, beyond the benefits that training programs can generate in social skills, they have not always been accompanied by clear evidence of their effectiveness and studies on the subject do not yet provide conclusive evidence. It is not enough to design, implement and publish educational programs aimed at developing interpersonal competencies. It is also necessary to evaluate these interventions, both to obtain empirical data on their greater or lesser degree of validity and to detect those aspects of such interventions that could be improved (Rubiales et al., 2018; Erçevik and Köseoglu, 2020; Tahan et al., 2020; Ramdhonee-Dowlot et al., 2021).

Consequently, the objective of this paper is to present the results of a pilot study on the application of the Interpersonal Skills Training Program for Adolescents (PEHIA), a program that has been published for several years and for which there are still no studies analyzing its preventive efficacy. Thus, we propose this research to resolve this situation.

## Method

### Sample

The participants were from two schools in the province of Salamanca (Spain). In both schools there were two groups of students in each course level, with approximately 30 students each. In the first, there were 61 ESO^3^ students (compulsory secondary school—USA seventh and eighth graders) and in the second there were 64 Bachillerato students (highschool—USA eleventh graders). All were contacted, and a total of 54 students decided to follow the program, 28 were ESO students and 26 studied Bachillerato. The inclusion criterion was to be willing to attend all the sessions that make up the program. Three ESO participants who had followed the program did not attend the second evaluation session. Thus, our final sample was 51 participants: 25 ESO (14 males and 11 females) and 26 Bachillerato (18 males and 8 females) students. It is a convenience sample obtained for a preliminary study.

### Instruments

The instruments used to collect data are standardized questionnaires with proven validity and reliability in different contexts and situations and with extensive publication in the scientific literature on this subject (Carmona and López, 2015).

#### Evaluation Instruments

*Social Anxiety Scale for Adolescents (“Escala de Ansiedad Social para Adolescentes” in Spanish: SAS-A; La Greca and López*, *1998**)*.

This questionnaire assesses social anxiety responses in adolescents in the context of their interpersonal relationships. It consists of 22 items that are scored on a five-point scale (1 = never; 5 = always). The 22 items are divided into three subscales: Fear of negative evaluation (FNE), Anxiety and social avoidance in New situations or in front of strangers (SAD-N) and Anxiety and social avoidance in front of people in General (SAD-G). Scores can be obtained either from the sum of the values of the items constituting each subscale or a total score can be obtained from the sum of all items except the four neutral items. High scores indicate high levels of anxiety. According to a study in Spanish population carried out by Olivares et al. (2005), it presents satisfactory internal consistency levels, calculated with Alpha coefficient (Cronbach, 1951). The internal consistency values were: FNE α =0.94, SAD-N α =0.87, and SAD-G α =0.8. According to this same study, the concurrent and discriminant validity of the questionnaire has been shown to be adequate, since the correlations with other measures of social anxiety are positive and statistically significant (Inglés et al., 2001).

*Adolescent Interpersonal Difficulties Assessment Questionnaire (“Cuestionario de Evaluación de Dificultades Interpersonales en la Adolescencia“ in Spanish: CEDIA; Inglés et al.*, *2003**)*.

This is a questionnaire for adolescents between 12 and 18 years of age. There are separate versions for males and females, identical except for the gender of nouns and pronouns. It is composed of 36 items distributed in five subscales: Assertiveness (AS), Heterosexual Relationships (RH), Public Speaking (HP), Family Relationships (RF) and Friends (AM). Each of the items is answered on a 5-point Likert scale, indicating how difficult each social situation and relationship generally is for the individual (0 = no difficulty; 4 = maximum difficulty). It is possibile to obtain a score for each subscale and a total score. The higher the score, the greater the interpersonal difficulty or social discomfort. The internal consistency coefficients (Cronbach's alpha) are: α = 0.90 (CEDIA), α = 0.83 (Assertiveness), α = 0.85 (Heterosexual Relationships), α = 0.75 (Public Speaking), α = 0.67 (Family Relationships), and α = 0.57 (Friends). The test-retest reliability, calculated using Pearson's product-moment $r_{\text{x}{\text{x}}^{\text{′}}}$ coefficient for a 2-week interval, is: CEDIA $r_{\text{xx}}^{\text{′}}$ =0.78, Assertiveness $r_{\text{xx}}^{\text{′}}$ =0.74, Heterosexual Relationships $r_{\text{xx}}^{\text{′}}$ =0.79, Public Speaking $r_{\text{xx}}^{\text{′}}$ =0.76, Family Relationships $r_{\text{xx}}^{\text{′}}$ =0.43, and Friends $r_{\text{xx}}^{\text{′}}$ =0.56 (Inglés et al., 2003). According to Inglés et al. (2001, 2003), it adequately discriminates adolescents with and without generalized anxiety, with and without fear of public speaking, introverted and extraverted, and emotionally stable and unstable.

#### Intervention Program

The Interpersonal Skills Training Program for Adolescents (PEHIA) program by Inglés et al. (2003) was applied. According to the author, the program is aimed at adolescents between 12 and 18 years of age with no apparent problems, or with internalizing problems, or with externalizing problems and in situations of social risk. It is a direct and systematic teaching program of interpersonal skills whose general objectives are to promote adequate interpersonal relationships and prevent maladaptive problems, as well as to eliminate and/or reduce possible interpersonal difficulties during adolescence. The specific objectives of the program are: to learn what interpersonal skills are; to become aware of the importance of this type of behavior; to differentiate shy, skillful, and aggressive styles; to acquire/improve the ability to express annoyance, dislike and/or displeasure; to acquire/improve the ability to refuse or say no, know which personal rights are, and adequately and effectively defend this personal rights; acquire/improve skills in flirting, introducing oneself and dating, make and accept compliments, initiate, maintain and end conversations; and develop negotiation skills necessary to reach agreements with parents.

These skills are taught over eight sessions of approximately 1 h duration, in a group setting. The cognitive-behavioral techniques are: Verbal instruction, modeling, behavioral rehearsal, positive feedback and reinforcement, and homework assignments.

### Design

The criterion variables were observed before and after the implementation of the treatment, and two comparison groups were used, secondary and high school students, thus creating a mixed factorial design, with the pre- and post-treatment measures as a within-subject variable and the academic groups as a between-subject variable. This type of design, one of the most used in basic and applied research in psychology (Kirk, 2013; Maxwell et al., 2018), is derived naturally of our objectives: to evaluate the effect of the intervention program (PEHIA) and to check whether this effect is similar in the two academic groups considered. As as stated at the end of the sampling subsection, these groups constituted a convenience sample obtained for a preliminary study.

### Procedure

Once permission had been requested from the bioethics committee of the University of Salamanca, the management teams of the two schools in Salamanca were informed of the existence of the program and the possibility of implementing it. After agreeing to collaborate in the study, the program was offered to students in the first and second year of Compulsory Secondary Education and the first year of Baccalaureate. They were informed of the objectives of the program and of the voluntary nature of participation in tutoring schedules.

As mentioned earlier in the participants section, a total of 28 secondary school and 26 high school students volunteered. Three students with incomplete data were dropped from the sample, leaving a total of 51 participants. Groups of eight to ten students were formed according to their tutoring schedules. All of them signed the informed consent form. The program was applied by a previously trained psychologist. The pre-treatment assessment was conducted two days before the first session. The post-treatment evaluation was conducted one week after the end of the last session of the program.

### Statistical Analysis

Data were analyzed and results were double checked using IBM-SPSS-26 and Jamovi-1.6 (based on R version 4.0), with the packages “afex” (Singmann, 2018) and “emmeans” (Lenth, 2020). Analyses of variance for mixed factorial designs were performed for the main scales and their subscales. In general, all data variables meet the assumptions satisfactorily, with minor deviations from homogeneity of variances in two subscales of the CEDIA questionnaire with less variability and scarce values (Family Relationships and Friends). Furthermore, in these scales, no effects of the intervention are found, as summarized below.

It is worth noting that the standard deviations in the interpersonal dificulties subscales are high relative to the means, which points to asymmetries and ill conditioned distributions. Robust analysis was performed, and the results were consistent with the results obtained with conventional parametric statistics. We used the R package WRS (Wilcox and Schönbrodt, 2017; Mair and Wilcox, 2020) functions “bwtrim” for trimmed means on each variable and also bootstrapped M-Estimators with the functions “sppba” for the group factor, “sppbb” for the treatment factor and “sppbi” for the interaction effect. The results agreed with those obtained with parametric ANOVA. The program output was a little bit cumbersome, so we decided the shortcut of approximating paired samples robust *t* tests (Yuen, 1974) on 20% trimmed means with Jamovi complement “Walrus” (Love and Mayr, 2018), based on WRS R package. Results are summarized in Table 1.

**Table 1:** Robust t statistics for SAS and CEDIA subscales main effects before and after treatment.

**Scale**	***M*_diff._**	***CI*_Lower_**	***CI*_Upper_**	**Yuen *t***	***p***	***d***
SAS total	7.55	3.46	11.64	3.77	<0.001	0.38
SAS FNE	3.00	1.00	5.03	3.02	0.005	0.34
SAS SAD-N	3.00	1.51	4.49	4.12	<0.001	0.44
SAS SAD-G	1.10	0.18	2.02	2.439	0.021	0.25
Cedia total	13.77	5.78	21.77	3.52	0.001	0.36
Cedia AS	6.74	2.60	10.88	3.33	0.002	0.33
Cedia RH	5.87	3.39	8.36	4.82	<0.001	0.40
Cedia HP	1.71	0.19	3.23	2.30	0.029	0.25
Cedia RF	0.52	−0.44	1.47	1.10	0.280	0.14
Cedia AM	0.06	−0.98	1.12	0.13	0.90	0.02

*SAS subscales: SAS Subscales; FNE, Fear of negative evaluation; SAS SAD-N, Anxiety and social avoidance in new situations or in front of strangers; SAS SAD-G, Anxiety and social avoidance in front of people in general*.

*Cedia AS, Assertivenes; Cedia RH, Heterosexual relationships; Cedia HP, Public Speaking; Cedia RF, Family; Cedia AM, Friends; M_diff_, Mean difference; CI_Lower_- CI_Upper_, 95% confidence interval of M_diff_; d, Cohen's d*.

The course groups in the ESO and Bachillerato Education in Spain correspond exactly with age groups so the age was actively controled by design. The ESO students were 12–13 years old (they are included in the courses by the year they were born), and the Bachillerato students were all 16 years old. When the three age groups were included, there were no statistically significant differences between participants with 12 or 13 years in the ESO group.

The gender variable was controlled but the analysis showed that its effects and interactions were negligible (η^2^_p_ between <0.001 and 0.077) except for the interaction with the treatment in the SAS SADN subscale (η^2^_p_ = 0.093, *p* = 0.033). We considered this result with caution: the η^2^ value (proportion of variance) was 0.01, so it was a very questionable effect and was arguable to take it seriously. All these results inclined us not to consider the variable “Género” (Gender) in the final report.

In the interpretation of the results, we follow the neo-Fisherian perspective in null hypothesis significance testing (Haig, 2017), paying more attention to the *p*-values than to whether or not they exceed a value that allows putting one, two, or three asterisks next to them. Nevertheless, we report the *p*-values as they appear in the outputs of the statistical programs. Furthermore, we prefer to look at the magnitude effects when they are clearly interpretable. Also, we have avoided the classic “t-shirt effect sizes” guidelines for the effect sizes [Glass et al., 1981; Ellis, 2010 (cited by Ellis, 2010, p. 104); Kline, 2020; among others]. We prefer a conceptual or substantive interpretation of size or magnitude effects based on their size (eta squared is simply a coefficient of determination, a proportion of variance) rather than on conventional guidelines.

Regardless, Cohen (1988) proposed for *r*^2^ (analog to η^2^ when there are only two groups) approximate values of 0.01 for small effects, 0.09 for medium effects, and 0.25 for large effects, with cutpoints eventually valid for η^2^, but not for η^2^_p_ (Pierce et al., 2004). The different partial eta squared (η^2^_p_) are not comparable (they are calculated with different denominators) even in the same experiment. There are also problems associated to effect sizes interpretation (Kelley and Preacher, 2012) not addressed here.

## Results

### Sample Description

The sample was distributed by gender with 19 females and 32 males, aged between 12 and 16 years (*M*_age_ = 14.3; *SD* = 1.96). By groups and gender, the ages of ESO females (*M*_age_ = 12.5; *SD* = 0.52) and ESO males (*M*_age_ = 12.3; *SD* = 0.47) were homogeneous as expected. Likewise the ages of Bachillerato females (*M*_age_ = 16.1; *SD* = 0.35) and Bachillerato males (*M*_age_ = 16.2; *SD* = 0.38) were very similar.

### Social Anxiety Responses (SAS)

The scores in the full social anxiety scale after the treatment were significantly lower (*M*_*post*_ = 37.6, *SD* = 10.10) than before treatment (*M*_*pre*_ = 44.67, *SD* = 12.50), [*F*_(1,49)_ = 23.11, *p* < 0.001, η^2^ = 0,09, η^2^_p_ = 0.32]. Participants scores were not significantly different across courses (*M*_*Bach*_ = 42.71, *SD* = 12.13; *M*_*ESO*_ = 39.46, *SD* = 11.46), [*F*_(1,49)_ = 1,32, *p* = 0.254, η^2^ = 0.019, η^2^_p_= 0.026]. There was no significant interaction between social anxiety and academic course, [*F*_(1,49)_ = 0.064] (values of *F* below 1 corresponds always to *p* > 0.5, as is well-known). This pattern was the same in all the social anxiety subscales; statistically significant main effects of the treatment implementation but no statistically significant effect of the academic groups nor interaction effect. Table 2 contains information only of the statistically significant main effects. The effect magnitude measures showed η^2^ medium to low effect sizes (proportions of total variance explained) and $\eta_{\text{p}}^{2}$ medium to high (proportions over the error) (Cohen, 1988, 1992).

**Table 2:** Statistics for SAS subscales before and after treatment.

**Scale**	***M*_**pre**_**	**CI *M*_**pre**_**	***M*_**post**_**	**CI *M*_**post**_**	***F*_**(1,49)**_**	***p***	**η^**2**^**	** $\eta_{\text{p}}^{2}$ **
Sas FNE	20.51 (6.14)	18.8–22.2	17.39 (5.87)	15.7–19.1	20.23	<0.001	0.06	0.29
Sas SADN	16.04 (5.05)	14.8–17.3	13.12 (3.82)	11.9–14.13	19.39	<0.001	0.10	0.28
Sas SADG	8.12 (3.14)	7.3–8.9	7.06 (2.41)	6.3–7.8	7.72	0.008	0.04	0.14

*Standard deviations are presented in parentheses*.

*SAS FNE, Fear of negative evaluation; SAS SAD-N, Anxiety and social avoidance in new situations or in front of strangers; SAS SAD-G, Anxiety and social avoidance in front of people in general; Mpre, Mean before treatment; CI Mpre, 95% confidence interval of Mpre; Mpost, Mean after treatment; CI Mpost, 95% confidence interval of Mpost*.

### Adolescent Interpersonal Difficulties (CEDIA)

**Figure 1:**
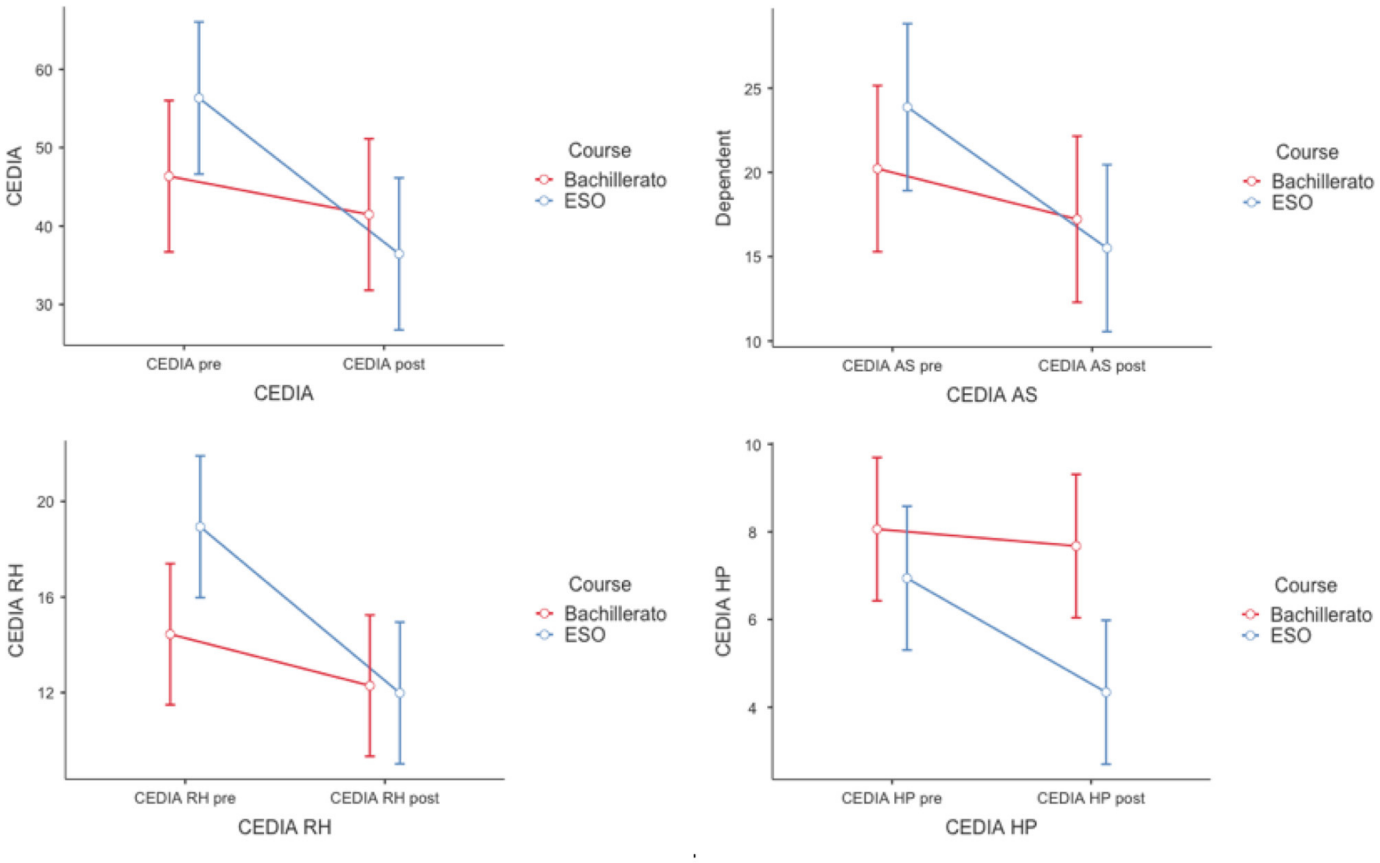
Interaction plots of the interpersonal dificulties scale and subscales. Interaction plot with 95% confidence intervals around the cell means. CEDIA scale interaction reported in the text; Cedia subscales. AS, Assertivenes, [*F*_(1,49)_ = 2.65, *p* = 0.11]; RH, Heterosexual relationships, [*F*_(1,49)_ = 7.31, *p* = 0.009, η^2^ = 0.02, η^2^_p_= 0.13]; HP, Public Speaking, [*F*_(1,49)_ = 4.20, *p* = 0.046, η^2^ = 0.02, η^2^_p_= 0.08]. The RF (family relationships) and AM (Friends) subscales are not shown for reasons stated in the text.

The scores in the full interpersonal dificulties scale after the treatment were significantly lower (*M*_*post*_ = 39.04, *SD* = 25.47) than before treatment (*M*_*pre*_ = 51.27, *SD* = 25.46), [*F*_(1,49)_ = 14.60, *p* < 0.001, η^2^ = 0,06, η^2^_p_ = 0.23]; participants scores were not significantly different across courses (*M*_*Bach*_ = 43.64, *SD* = 22.39; *M*_*ESO*_ = 46.42, *SD* = 28.08), [*F*_(1,49)_ = 0.167], but these results are qualified by a significant interaction, [*F*_(1,49)_ = 5.35, *p* = 0.025, η^2^ = 0.02, η^2^_p_ = 0.099]. This pattern was roughly the same in all the interpersonal dificulties subscales; statistically significant main effects of the treatment implementation (see Figure 1) but no statistically significant effect of the academic groups and a statistically significant interaction effect in some subscales. Table 3 contains information only of the statistically significant main effects. No statistically significant treatment effects nor interactions were found for the scales previously cited (RF and AM, nor the interaction in the AS subscale). The effect magnitude measures showed η^2^ medium to low effect sizes and $\eta_{\text{p}}^{2}$ medium to high (Cohen, 1988, 1992; Pierce et al., 2004). The interactions at the interpersonal dificulties scale (CEDIA) and subscales (AS = Assertivenes; RH = Heterosexual relationships; HP = Public Speaking) are depicted in Figure 1.

**Table 3:** Statistics for CEDIA subscales main effects before and after treatment.

**Scale**	***M*_**pre**_**	**CI *M*_**pre**_**	***M*_**post**_**	**CI *M*_**post**_**	***F*_**(1,49)**_**	***p***	**η^**2**^**	** $\eta_{\text{p}}^{2}$ **
Cedia AS	22.02 (13.03)	18.6–25.5	16.39 (11.91)	12.9–19.9	11.88	<0.001	0.05	0.20
Cedia RH	16.67 (7.81)	14.6–18.8	12.16 (7.37)	10.0–14.2	26.30	<0.001	0.08	0.35
Cedia HP	7.49 (4.60)	6.3–8.7	6.02 (3.97)	4.9–7.2	7.62	0.008	0.03	0.14
Cedia RF	2.48 (3.21)	1.7–3.3	1.90 (2.80)	1.1–2.7	1.41	0.241	0.01	0.03
Cedia AM	2.63 (2.73)	1.9–3.4	2.56 (2.82)	1.8–3.3	0.02	0.879	0.00	0.00

*Standard deviations are presented in parentheses*.

*Cedia subscales: AS, Assertivenes; Cedia RH, Heterosexual relationships; Cedia HP, Public Speaking; Cedia RF, Family; Cedia AM, Friends; M_pre_, Mean before treatment; CI M_pr__e_, 95% confidence interval of M_pre_; M_post_, Mean after treatment; CI M_post_, 95% confidence interval of M_post_*.

## Discussion

To our knowledge, this investigation is the first study to assess the effectiveness of the PEHIA, the Interpersonal Skills Training Program for Adolescents (Inglés, 2003; Inglés Saura, 2017).

The results obtained with the SAS-A questionnaire which measures social anxiety for adolescents, indicate that the PEHIA program reduced the level of anxiety presented in interpersonal relationships by the adolescents who participated in the study. This reduction was observed in both high school and secondary school students. Both the effect sizes and the confidence intervals of all the SAS scales support this find.

Regarding the degree of difficulty in establishing relationships with other people, as measured with the CEDIA questionnaire, the PEHIA program helped secondary school students to overcome this difficulty to a greater extent than high school students. Specifically, assertiveness, interpersonal relationships and public speaking have been improved, but no significant differences have been found in the difficulty experienced in family and friend relationships. This is probably due to the fact that before the application of the program, the difficulty scores on these two scales were low.

The results obtained in assertiveness, interpersonal relations and public speaking suggest that the PEHIA program was feasible and the results show promise in reducing anxiety.

Althoug, as we have already indicated we have not found studies that analyze the effectiveness of the PEHIA program, we can discuss our results with other similar investigations.

For example, Tahan et al. (2020) investigated the effect of communication skills training on social empowerment and social adjustment of so-called “slow-paced” adolescents (e.g., those who need psychological, physical, and emotional drivers to actualize their potential abilities). The results showed that communication skills training has a significant impact on social empowerment (*F* = 15.47, *p* = 0.001) and social adjustment (*F* = 49.64, *p* = 0.001). In other words, the communication skills training impacts on social empowerment and its components as well as social maturity.

van Loon et al. (2019) investigated the effectiveness of school-based skills-training programs social to promote the mental health of adolescents. These authors demonstrated reduced stress levels, reduced internalizing and externalizing behavior, increased self-esteem and improved well-being in adolescents who received this type of program.

On the other hand, Ramdhonee-Dowlot et al. (2021) examined the effectiveness of a transdiagnostic prevention program, Super Skills for Life (SSL), among children and adolescents with emotional problems in residential care institutions. SSL is based on the principles of cognitive behavior therapy, behavioral activation, social skills training, and uses video-feeback and cognitive prepartion as part of the treatment. Children and adolescents participants showed significant improvements in internalizing symptoms (e.g., anxiety and depression), externalizing symptoms (e.g., conduct problems and hyperctivity), and inhibitory control, an increase in adaptive and decrease in a maladaptive emotion regulation strategies, at both post-intervention and follow-up.

Olivares-Olivares et al. (2019) studied the role and effects of a Social Skills Training (SST) frequently included in the treatment of social anxiety disorder (SAD). These authors found that participants who were trained in the SST obtained better results in the post-test and follow-ups, as well as a lower dropout rate (6:1). Concluding that the use of SST reduces the dropout rate of treated adolescents and increases the effectiveness of the intervention program for adolescents with socia phobia.

## Conclusion

Given that the aim of this study was to provide evidence regarding the degree of efficacy of the PEHIA program and since the results obtained are encouraging, it would be advisable, as future research, to carry out large-scale research to provide data that can inform on its effects. If, in a study with a larger number of participants similar results are obtained, this would provide us with a scientifically validated program that has been published in Spain for years. Also, it would be one more step toward meeting the requirements established by the American Psychological Association (APA) to validate programs.

Some of the limitations of this research would be related, on the one hand, to the lack of subjective information (qualitative data on the personal experience of the participants), however, the verbal information (feedback) that they gave were que loved it. On the other hand, with the loss of participants and experiments with small sample. The groups in bigger sizes can give us powerful results, however, participants loss is a common problem in psychological studies.

Finally, our research is a preliminary study, and its intentios is to serve as the first evidence for its possible implementation on a larger scale. Of course, more evidence is needed for this, but our results are a first step in that direction.

Anyway, the PEHIA program are easily implemented and the training of the behavior modification psychologist who will implement it is not difficult or time consuming.

## Data Availability Statement

The raw data supporting the conclusions of this article will be made available by the authors, without undue reservation.

## Ethics Statement

The studies involving human participants were reviewed and approved by Comité de Bioética de la Universidad de Salamanca. Written informed consent to participate in this study was provided by the participants' legal guardian/next of kin.

## Author Contributions

IS-P: conceptualization, methodology, investigation, writing-original draft preparation, writing—review and editing, and supervision. JD-S-M: methodology, formal analysis, software, data curation, and supervision. M-CE-L: conceptualization, writing-original draft preparation, writing—review and editing, and supervision. All authors contributed to the article and approved the submitted version.

## Conflict of Interest

The authors declare that the research was conducted in the absence of any commercial or financial relationships that could be construed as a potential conflict of interest.

## Publisher's Note

All claims expressed in this article are solely those of the authors and do not necessarily represent those of their affiliated organizations, or those of the publisher, the editors and the reviewers. Any product that may be evaluated in this article, or claim that may be made by its manufacturer, is not guaranteed or endorsed by the publisher.

## Footnotes

^2^ISTPA would be the acronym in English, but we keep the original acronym PEHIA from the original Spanish study (Inglés, 2003) presented in the abstract.

^3^In the spanish educational curriculum, ESO is the acronim for Educación Secundaria Obligatoria (Compulsory Secondary Education), divided in 4 courses.
